# MolTrans: Molecular Interaction Transformer for drug–target interaction prediction

**DOI:** 10.1093/bioinformatics/btaa880

**Published:** 2020-10-18

**Authors:** Kexin Huang, Cao Xiao, Lucas M Glass, Jimeng Sun

**Affiliations:** Health Data Science, Harvard University, Boston, MA 02120, USA; Analytics Center of Excellence, IQVIA, Cambridge, MA 02139, USA; Analytics Center of Excellence, IQVIA, Cambridge, MA 02139, USA; Department of Computer Science, University of Illinois at Urbana-Champaign, Urbana, IL 61801, USA

## Abstract

**Motivation:**

Drug–target interaction (DTI) prediction is a foundational task for *in-silico* drug discovery, which is costly and time-consuming due to the need of experimental search over large drug compound space. Recent years have witnessed promising progress for deep learning in DTI predictions. However, the following challenges are still open: (i) existing molecular representation learning approaches ignore the sub-structural nature of DTI, thus produce results that are less accurate and difficult to explain and (ii) existing methods focus on limited labeled data while ignoring the value of massive unlabeled molecular data.

**Results:**

We propose a Molecular Interaction Transformer (MolTrans) to address these limitations via: (i) knowledge inspired sub-structural pattern mining algorithm and interaction modeling module for more accurate and interpretable DTI prediction and (ii) an augmented transformer encoder to better extract and capture the semantic relations among sub-structures extracted from massive unlabeled biomedical data. We evaluate MolTrans on real-world data and show it improved DTI prediction performance compared to state-of-the-art baselines.

**Availability and implementation:**

The model scripts are available at https://github.com/kexinhuang12345/moltrans.

**Supplementary information:**

Supplementary data are available at *Bioinformatics* online.

## 1 Introduction

Drug discovery is notoriously costly and time-consuming due to the need of experimental search over large drug compound space. Drug–target protein interaction (DTI) prediction serves as the foundation for finding new drugs (i.e. virtual screening) and new indications of existing drugs (i.e. drug repositioning), since the therapeutic effects of drug compounds are detected by examining DTIs (Hughes *et al.*, 2011). During the compound identification process, researchers often need to conduct assay experiments and search over 97 M possible compounds in a candidate database (Broach *et al.*, 1996).

Luckily, with massive biomedical data and knowledge being collected and available, along with the advances of deep learning technologies which demonstrated huge success in many application areas, the drug discovery process particularly DTI prediction has been significantly enhanced. Recently, various deep models have shown encouraging performance in DTI predictions. They often take drug and protein data as inputs, cast DTI as a classification problem, and make prediction by feeding the inputs through deep learning models such as deep neural network (DNN) (Unterthiner *et al.*, 2014), deep belief network (DBN) (Wen *et al.*, 2017) and convolutional neural network (CNN) (Mayr *et al.*, 2018; Öztürk *et al.*, 2018, 2019). Despite these efforts, the following challenges are still open.

1. **Inadequate modeling of interaction mechanism**. Existing works (Gao *et al.*, 2018; Öztürk *et al.*, 2018, 2019) learn molecular representation and make prediction based on whole molecular structure of drugs and proteins, ignoring that the interactions are sub-structural—only involving relevant sub-structures of drugs and proteins (Jia *et al.*, 2009; Schenone *et al.*, 2013). The full-structural molecular representations introduce noises and affect the prediction performance. Also, the learned representations are hard to interpret since they do not provide a tractable path to indicate which sub-structures of drugs and proteins contribute to the interactions.
2. **Restricted to limited labeled data**. Previous works (Gao *et al.*, 2018; Lee *et al.*, 2019; Öztürk *et al.*, 2018, 2019; Wen *et al.*, 2017) focus on data in hand and limit the scope within several thousands of drugs and proteins while ignoring the vast (e.g. order of millions) unlabeled biomedical data available. The model architectures in previous works are also not designed to enable the integration of massive dataset.

**Present Work**. To solve these challenges, we propose a transformer (Vaswani *et al.*, 2017)-based bio-inspired molecular data representation method [coined as Molecular Interaction Transformer (MolTrans)] to leverage vast unlabeled data for *in-silico* DTI prediction. MolTrans is enabled by the following technical contributions:

1. **Knowledge inspired representation and interaction modeling for more accurate and explainable prediction**. Inspired by the knowledge that DTI is sub-structural, MolTrans derives a data-driven method called Frequent Consecutive Sub-sequence (FCS) mining that is adaptable to extract high-quality fit-sized sub-structures for both protein and drug. In addition, MolTrans includes a bio-inspired interaction module imitating the real biological DTI process. The new sub-structure fingerprints enable a tractable path for understanding which sub-structure combination has more relevance to the outcome through an explicit map in the interaction module.
2. **Leverage massive unlabeled biomedical data**. MolTrans mines through millions of drugs and proteins sequences from multiple unlabeled data sources to extract high-quality sub-structures of drugs and proteins. The vast data result in a much higher quality sub-structures than using small training dataset alone. We also augment the representation using transformers (Vaswani *et al.*, 2017), which captures the complex signals among the large sequential sub-structures outputs generated from the unlabeled data.

We provide a comprehensive performance comparison of state-of-the-art methods on various realistic drug discovery settings include unseen drug/target problems and in scarce training dataset setup. We show empirically that MolTrans has robust improved predictive performance over state-of-the-art baselines by up to 25% over the best performing baseline.

**Related work**. Numerous computational methods have been developed for DTI prediction problem. Similarity-based methods such as kernel regression (Pahikkala *et al.*, 2015) and matrix factorization (Zheng *et al.*, 2013) methods exploit known DTI’s drug–target similarity information and infer new ones. However, these methods are shown to be not generalizable to different protein classes (Wen *et al.*, 2017). Feature-based methods feed numerical descriptors of drug and proteins into downstream prediction models. Popular numerical descriptors include ECFP (Rogers and Hahn, 2010) and PubChem (Bolton *et al.*, 2008) for drugs, Composition–Transition–Distribution (CTD; Dubchak *et al.*, 1995) and protein sequence composition descriptor (PSC; Cao *et al.*, 2013) for proteins. Classic machine learning methods such as gradient boosting (He *et al.*, 2017) have shown promises in predictive performance. Recently, deep learning-based methods (Öztürk *et al.*, 2019; Tsubaki *et al.*, 2019; Unterthiner *et al.*, 2014; Wen *et al.*, 2017) have shown further improvement of performance due to its capability to capture complex non-linear signals of DTI. MolTrans differs from existing works with (i) its knowledge-driven model architecture design rather than direct application of existing deep learning models; (ii) emphasis on interpretability instead of predictive performance alone to potentially aid medical chemists for better decision making and (iii) usage of external drug and target data to complement interaction dataset.


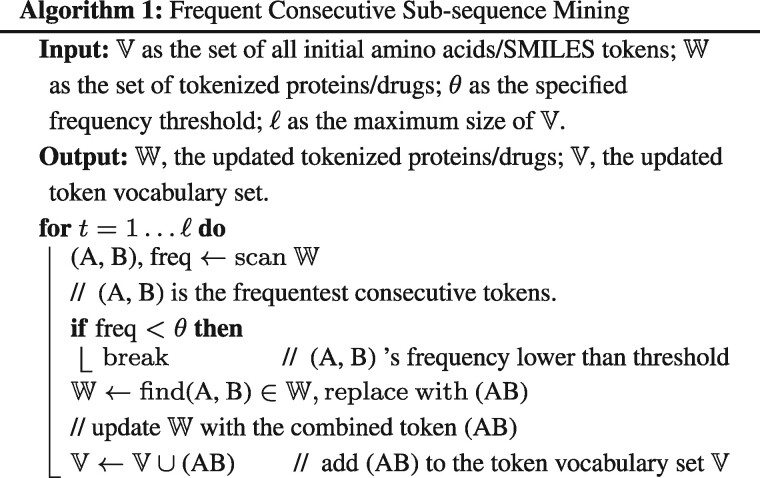


## 2 Materials and methods

### 2.1 Problem definition

We formulate the DTI prediction as a classification task to determine whether a pair of drug and target protein will interact. In our setting, drug is represented by the Simplified Molecular Input Line Entry System (SMILES) $S_{i}$, which consists of a sequence of chemical atoms and bonds tokens (e.g. C, O, S), generated by depth-first traversal over the molecule graph. We denote **S** for drug’s SMILES representation. Target protein, denoted as **A**, is represented by a sequence of protein tokens, where each token is one of the 23 amino acids. The notation table is provided in Supplementary Material S1. The DTI prediction task is defined as below.

Problem 1 (**DTI Prediction**). Given compound sequence $S=\{S_{1},\ldots ,S_{n}\}$ for *n* drugs and protein sequence $A=\{A_{1},\ldots ,A_{m}\}$ for *m* proteins, the DTI prediction task can be casted as to learn a function mapping F:S×A→[0,1] from drug–target pairs to an interaction probability score.

### 2.2 The MolTrans method

**Fig. 1.**
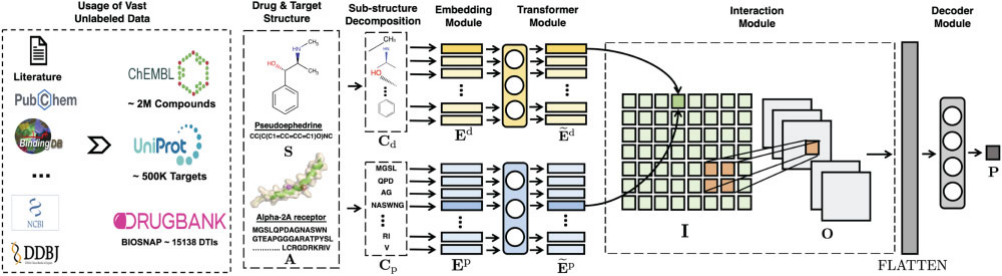
MolTrans workflow: (**a**) MolTrans utilizes vast unlabeled data. (**b**) Given the input pair of drug **S** and protein **A**, MolTrans extracts a sequence of sub-structures $C_{d}$ and $C_{p}$ via Algorithm 1. (**c**) Each sub-structure is embedded into a latent feature vector $E^{d}$ and $E^{p}$ through a learnable embedding table via Equation (1). Then, drug/protein sequence of sub-structure embedding is fed into drug/target transformer encoders, respectively, to obtain an augmented contextual representation ${\tilde{E}}^{d}$ and ${\tilde{E}}^{p}$ via Equation (2). (d) An interaction map **I** measuring interaction intensity among sub-structures is generated via Equation (3). The interaction is further optimized by a CNN layer that models higher-order interaction, which results in a tensor **O** via Equation (4). (e) A decoder module then feed the tensor for a classifier to output the DTI probability **P** via Equation (5). All modules are trained end-to-end with the binary classification loss via Equation (6)

The MolTrans framework learns to predict DTI as follows. Given the input drug and protein data, a FCS mining module first decomposes them into a set of explicit sequences of sub-structures using a specialized decomposition algorithm. The outputs are then fed into a augmented transformer embedding module to obtain an augmented contextual embedding for each sub-structure through transformer encoders (Vaswani *et al.*, 2017). Next, in the interaction prediction module, drug sub-structures are paired with protein sub-structures with pairwise interaction scores. A CNN layer is later applied on the interaction map to capture higher-order interactions. Finally, a decoder module outputs a score indicating the probability of pairwise interactions. Method illustration is provided in Fig. 1.

#### 2.2.1 FCS mining module

Driven by the domain knowledge that DTI happens in a sub-structural level, MolTrans first decomposes molecular sequence for proteins and drugs into sub-structures. In particular, we propose a data-driven sequential pattern mining algorithm called FCS algorithm to find recurring sub-sequences across drug and protein databases. Inspired by the invention of sub-word units in the natural language processing field (Gage, 1994; Sennrich *et al.*, 2015), FCS aims to generate a set of hierarchy of frequent sub-sequences for sequences.

The algorithm is summarized in Algorithm 1. FCS decomposes each sequence of protein/drug hierarchically into sub-sequences, smaller sub-sequences and individual atoms/amino acids symbols. FCS first initializes a vocabulary set V of distinctive amino acid tokens or SMILES strings characters and given the tokens, tokenizes the entire drug/protein corpus. We call the tokenized set W. Then, it scans through W and identifies the most frequent consecutive tokens (A, B). FCS then updates every (A, B) in the tokenized set W with the new token (AB) and also adds this new token to the vocabulary set V. Then this process of *scan*, *identify*, *update* is repeated until no frequent token is above the threshold *θ* or the size of V reaches a pre-defined maximum value ℓ. Through this operation, frequent sub-sequences are merged into one token and sub-sequences that are not frequent enough are decomposed into a set of shorter tokens. In the end, for a drug/protein, FCS results in a sequence $C=\{C_{1},\ldots ,C_{k}\}$ of sub-structural drug/target proteins with size of *k*, where each *C_i_* is from the set V.

Using FCS algorithm, MolTrans converts input drug and target to a sequence of explicit sub-structures $C_{d}$ and $C_{p}$, respectively. The significance of FCS is threefolds:

1. It distinguishes from previous sub-structure fingerprinting methods as it is more explainable. Explicit sub-structure fingerprint such as PubChem encoding has on average 100 granular sub-structures for a small molecule where many sub-structures are a subset of other ones, making it intractable to know which sub-structure leads to the outcome. In contrast, FCS drug encoding is capable of giving explicit hints as it decomposes each drug molecule into discrete and moderate size *partitions* of sub-structures as shown in Section 3.7. It allows for leveraging the massive unlabeled data for improved sub-structure mining. For example, we use the Uniprot dataset (Boutet *et al.*, 2007) consists of 560 823 unique protein sequences and the ChEMBL database (Gaulton *et al.*, 2012) which includes 1 870 461 drug SMILES strings. We observe that the quality of the mined sub-structures originates from the massive unlabeled data we used. In small datasets, the occurrences of many useful sub-structures are below the reasonable minimum frequency whereas a large aggregation dataset can successfully identify them with a larger sequences pool. We also show that the encoding has better predictive power when using massive unlabeled data compared to using small datasets, in Section 3.8.
2. FCS can capture fundamental and meaningful biomedical semantics. The generated sub-structures are associated with fundamental unit of drugs and proteins that recur frequently. We find that the FCS algorithm identify similar set of fundamental biochemical sub-structures given different dataset characteristics such as different types of organisms of the protein dataset and the drug-likeliness of drug dataset, suggesting the robustness of FCS algorithm (Supplementary Material S1). In general, we apply a more general dataset (e.g. ChEMBL) instead of a focused dataset (e.g. approved drugs in DrugBank) because larger dataset can improve downstream predictive performance (Section 3.8).

#### 2.2.2 Augmented transformer embedding module

To capture the chemical semantics of sub-structures, MolTrans includes an augmented embedding module where it first initializes a learnable sub-structure lookup dictionary and then augment the embedding with the contextual sub-structural information via transformer encoders (Vaswani *et al.*, 2017). The transformer is a state-of-the-art deep learning architecture that leverages self-attention mechanism to generate contextual embedding. It was originally developed to natural language processing tasks. Here, we adapted it for molecule representation learning. In our setting, the self-attention mechanism in the transformer encoder modifies each input sub-structure embedding by learning from all the sub-structures from the same molecule. The resulting sub-structural embedding is better because it is contextual by taking account into the complex chemical relationships among the neighboring sub-structures.

Concretely, for each input drug–target pair, we transform the corresponding sequence of sub-structures $C_{p}$ and $C_{d}$ into two matrices $M^{p}\in {\mathbb{R}}^{k\times \Theta_{p}}$ and $M^{d}\in {\mathbb{R}}^{l\times \Theta_{d}}$, where *k*/*l* is the total size of sub-structures for drug/protein or the cardinality of the vocabulary set V from FCS algorithm, Θ_*p*_ and Θ_*d*_ are the maximum lengths of sub-structure sequences for protein and drug, and each column $M_{i}^{p}$ and $M_{j}^{d}$ is a one-hot vector corresponding to the sub-structure index for the *i*th sub-structure of protein sequence and *j*th sub-structure of drug sequence. The content embedding $E_{{\mathrm{cont}}_{i}}^{p},E_{{\mathrm{cont}}_{j}}^{d}$ for each protein and drug is generated via a learnable dictionary lookup matrix $W_{\text{cont}}^{p}\in {\mathbb{R}}^{\vartheta \times k}$ and $W_{\text{cont}}^{d}\in {\mathbb{R}}^{\vartheta \times l}$ such that

$$E_{{\mathrm{cont}}_{i}}^{p}=W_{\text{cont}}^{p}M_{i}^{p},\;E_{{\mathrm{cont}}_{j}}^{d}=W_{\text{cont}}^{d}M_{j}^{d},$$

where ϑ is the size of latent embedding for each sub-structure.

Since MolTrans uses sequential sub-structures, we also include a positional embedding $E_{{\mathrm{pos}}_{i}}^{p},E_{{\mathrm{pos}}_{j}}^{d}$ via a lookup dictionary (Vaswani *et al.*, 2017) $W_{\text{pos}}^{p}\in {\mathbb{R}}^{\vartheta \times \Theta_{p}}$ and $W_{\text{pos}}^{d}\in {\mathbb{R}}^{\vartheta \times \Theta_{d}}$:

$$E_{{\mathrm{pos}}_{i}}^{p}=W_{\text{pos}}^{p}I_{i}^{p},\;E_{{\mathrm{pos}}_{j}}^{d}=W_{\text{pos}}^{d}I_{j}^{d},$$

where $I_{i}^{p}\in {\mathbb{R}}^{\Theta_{p}}/I_{j}^{d}\in {\mathbb{R}}^{\Theta_{d}}$ is a single hot vector where *i*/*j*th position is 1.

The final embedding $E_{i}^{p},E_{j}^{d}$ are generated via the sum of content and positional embedding:

(1) $$E_{i}^{p}=E_{{\mathrm{cont}}_{i}}^{p}+E_{{\mathrm{pos}}_{i}}^{p},E_{j}^{d}=E_{{\mathrm{cont}}_{j}}^{d}+E_{{\mathrm{pos}}_{j}}^{d}.$$

The models above outputs a set of independent sub-structure embedding. However, these sub-structures have chemical relationships (e.g. Octet rules) among themselves to capture these contextual information, we further augment the embedding using a transformer encoder layers (Vaswani *et al.*, 2017):

(2) $${\tilde{E}}^{p}={\text{Transformer}}_{\text{Protein}}(E^{p}),{\tilde{E}}^{d}={\text{Transformer}}_{\text{Drug}}(E^{d}).$$

#### 2.2.3 Interaction prediction module

MolTrans includes an interaction module that consists of two layers: (i) an interaction tensor to model pairwise sub-structural interaction and (ii) a CNN layer over interaction map to extract neighborhood interaction.

**Pairwise interaction**. To model the pairwise interaction, for each sub-sequence *i* in protein and sub-sequence *j* in drug, we have

(3) $$I_{i,j}=F({\tilde{E}}_{i}^{p},{\tilde{E}}_{j}^{d}),$$

where F is a function that measures the interaction between the pairs. It can be any function such as sum, average and dot product. Therefore, after this layer, we have a tensor $I\in {\mathbb{R}}^{\Theta_{d}\times \Theta_{p}\times \Phi }$, where $\Theta_{d}/\Theta_{p}$ is the length of sub-sequences for drug/protein, respectively, and Φ is the size of the output of function F, where each column in this tensor takes account into the interaction of individual sub-structure of proteins and drugs. To provide explainability, we favor dot product as the aggregation function because it generates a single scalar that explicitly measures the intensity of interaction between individual target-drug sub-structural pair. As dot product output is one-dimensional for every pair, **I** becomes a two-dimensional interaction map. If a value in the map is high, it will be activated in the downstream layer and have a higher likelihood of DTI interaction. Through end-to-end learning, if a pair of sub-structures indeed interact, they will have high interaction score in the corresponding sub-structure pair position in the interaction map. Thus, by examining this map, we directly see which sub-structure pairs contribute to the final outcome.

**Neighborhood interaction**. Nearby sub-structure of proteins and drugs influence each other in triggering the interactions. Hence, besides modeling the individual pairwise interaction, it is also necessary to model the interaction to the nearby regions. We achieve this through a CNN (Krizhevsky *et al.*, 2012) layer on top of the interaction map **I**. The intuition is that by applying several order-invariant local convolution filters, interaction to nearby regions can be captured and aggregated. We obtain the output representation **O** of the input drug–target pair:

(4) O=CNN(I).

This interaction module is inspired from the Deep Interactive Inference Network (Gong *et al.*, 2018). Thanks to this explicit interaction modeling, we can later visualize the strength of individual sub-structural interaction pair from the interaction map. To output a probability indicating the likelihood of interaction, we first flatten the **O** into a vector and use a linear layer parameterized by weight matrix $W_{o}$ and bias vector $b_{o}$:

(5) $$P=\sigma (W_{o}\text{FLATTEN}(O)+b_{o}),$$

where $\sigma (a)=\frac{1}{1+\text{exp}\;(-a)}$.

The entire network with parameters $W_{\text{cont}}^{p},\;W_{\text{cont}}^{d},\;W_{\text{pos}}^{p},\;W_{\text{pos}}^{d}$$W_{o},\;b_{o}$, the transformer encoders weights and CNN weights can be jointly optimized through the binary classification loss:

(6) L=Y  log  (P)+(1−Y)  log  (1−P),

where **Y** is the ground truth label.

### 2.3 Implementation

MolTrans is implemented in PyTorch (Paszke *et al.*, 2019). For the FCS algorithm, we set the minimum number of occurrences of sub-structures in the dataset to be 500 for drugs and proteins, which results in 23 532 drug sub-structures and 16 693 protein sub-structures. For transformer encoders, we use two layers of transformer encoders for both drug and proteins. The input embedding is of size 384 and we set 12 attention heads for each transformer encoder with intermediate dimension 1536. We set the maximum length of sequence for drug to be 50 and protein to be 545 to cover 95% of them in the dataset. We cut/pad for the parts that are above/below the maximum length. We show that the model performance is not biased against sequence length in Supplementary Material S2. For the CNN, we use three filters with kernel size three. For optimization hyper-parameters, we use Adam optimizer with learning rate of 1e−5. We set the batch size to be 64 and we allow it to run for 30 epochs. It converges between 8 and 15 epochs. The dropout rate is 0.1.

## 3 Result

We design experiments to answer the following questions.

Q1: Does MolTrans improve DTI predictive performance?

Q2: How well does MolTrans tackle the unseen drug/target cases?

Q3: How does MolTrans respond to large number of missing data?

Q4: How does performance vary given different protein families?

Q5: Does MolTrans provide useful knowledge about DTI?

Q6: How does each component of MolTrans contribute to the predictive performance gain?

### 3.1 Experimental setup

**Dataset**. We use the MINER DTI dataset from BIOSNAP collection (Zitnik *et al.*, 2018a) as our main dataset of experiments. It consists of 4510 drug nodes and 2181 protein targets, and 13 741 DTI pairs from DrugBank (Wishart *et al.*, 2008). BIOSNAP dataset only contains positive DTI pairs. For negative pairs, we sample from the unseen pairs, following common practice (Zhang and Chen, 2018; Zitnik *et al.*, 2018b). We obtain a balanced dataset with equal positive and negative samples. In addition to BIOSNAP, we also include two benchmark datasets in the main predictive performance comparison experiment. DAVIS consists of wet lab assay *K*_d_ values among 68 drugs and 379 proteins (Davis *et al.*, 2011) and BindingDB consists of *K*_d_ values among 10 665 drugs and 1413 proteins (Liu *et al.*, 2007). DTI pairs that have *K*_d_ values <30 units are considered positive. For balanced training, we sub-sample the same number of negative DTI pairs as the positive samples for training set. We keep the dataset negative ratios in the validation and testing set. Dataset statistics are provided in Table 1.

**Table 1.** Dataset statistics

Dataset	# Drugs	# Proteins	# Pos Interactions	# Neg Interactions
BIOSNAP	4510	2181	9619/1374/2748	9619/1374/2748
DAVIS	68	379	1043/160/303	1043/2846/5708
BindingDB	10 665	1413	6334/927/1905	6334/5717/11 384

*Note*: For the number of interactions columns, we include training/validation/testing interactions statistics in onefold of data.

**Metrics**. We use ROC-AUC (area under the receiver operating characteristic curve) and PR-AUC (area under the precision–recall curve) as metrics to measure the binary classification performance. In addition, we use sensitivity and specificity metrics where the threshold is the one that has the best *F*1 score in the validation set.

**Evaluation strategies**. We divided the dataset into training, validation and testing sets in a 7:1:2 ratio. For every experiment, we conduct five independent runs with different random splits of dataset. We then select the best performing model based on ROC-AUC performance from the validation set. The selected model via validation is then evaluated on the test set with the result reported below.

**Technologies**. We use a server with 2 Intel Xeon E5-2670v2 2.5 GHz CPUs, 128 GB RAM and 2 NVIDIA Tesla P40 GPUs.

### 3.2 Baselines

We compared MolTrans with the following baselines. We focus on state-of-the-art deep learning models as they have demonstrated superior performance over shallow models.

1. **LR** (Cao *et al.*, 2013; Rogers and Hahn, 2010) applies a logistic regression model on the concatenated drug and protein feature vectors. We experiment on all the combinations for ECFP4 (Rogers and Hahn, 2010) and PubChem (Wang *et al.*, 2009) for drugs and PSC (Cao *et al.*, 2013) and CTD (Dubchak *et al.*, 1995) for proteins. We find ECFP4 for drugs and PSC for protein has the highest performance.
2. **DNN** uses a three layer DNN with hidden size 1024 on top of the ECFP4 and PSC concatenated vector.
3. **GNN-CPI** (Tsubaki *et al.*, 2019) uses graph neural network to encode drugs and use CNN to encode proteins. The latent vectors are then concatenated into a neural network for compound–protein interaction prediction. We follow the same hyper-parameter setting described in the paper.
4. **DeepDTI** (Wen *et al.*, 2017) models DTI using DBN (Hinton, 2009), which is a stack of Restricted Boltzmann Machines (Hinton, 2012). It uses the concatenation of ECFP2, ECFP4, ECFP6 as the drug feature and uses PSC for protein features. We optimize the hyper-parameters described from the paper based on validation set performance.
5. **DeepDTA** (Öztürk *et al.*, 2018) applies CNN on both raw SMILES string and protein sequence to extract local residue patterns. The task is to predict binding affinity values. We add a Sigmoid activation function in the end to change it to a binary classification problem and we conduct hyper-parameter search to ensure fairness.
6. **DeepConv-DTI** (Lee *et al.*, 2019) uses CNN and global max pooling layer to extract various length local patterns in protein sequence and applies fully connected layer on drug fingerprint ECFP4. It conducts extensive experiment on different datasets and is the state-of-the-art model in DTI binary prediction task. We follow the same hyper-parameter setting described in the paper.

### 3.3 Q1: MolTrans achieves superior predictive performance

To answer Q1, we randomly select 20% drug protein pairs as test set. Table 2 shows MolTrans has consistently better predictive baselines in the DTI prediction setting in ROC-AUC and PR-AUC across all datasets. MolTrans has up to 25% increase over best performing baseline (DAVIS PR-AUC). Note that due to different thresholds across different methods, the sensitivity and specificity may vary.

**Table 2.** Performance comparison (five random runs)

Method	ROC-AUC	PR-AUC	Sensitivity	Specificity	Threshold
Dataset 1: BIOSNAP					
LR	0.846±0.004	0.850±0.011	0.755±0.039	0.800±0.018	0.434
DNN	0.849±0.003	0.855±0.010	0.776±0.040	0.838±0.024	0.499
GNN-CPI	0.879±0.007	0.890±0.004	0.780±0.014	0.819±0.012	0.349
DeepDTI	0.876±0.005	0.876±0.006	**0.789±0.027**	0.845±0.017	0.347
DeepDTA	0.876±0.005	0.883±0.006	0.781±0.015	0.824±0.012	0.466
DeepConv-DTI	0.883±0.002	0.889±0.005	0.770±0.023	0.832±0.016	0.441
MolTrans	0.895±0.002	0.901±0.004	0.775±0.032	0.851±0.014	0.431
Dataset 2: DAVIS					
LR	0.835±0.010	0.232±0.023	0.699±0.051	0.842±0.033	0.399
DNN	0.864±0.009	0.258±0.024	0.764±0.045	0.860±0.038	0.489
GNN-CPI	0.840±0.012	0.269±0.020	0.696±0.047	0.842±0.039	0.487
DeepDTI	0.861±0.002	0.231±0.006	0.751±0.015	0.853±0.012	0.387
DeepDTA	0.880±0.007	0.302±0.044	0.764±0.045	0.865±0.020	0.482
DeepConv-DTI	0.884±0.008	0.299±0.039	0.754±0.040	0.880±0.024	0.438
MolTrans	0.907±0.002	0.404±0.016	0.800±0.022	0.876±0.013	0.447
Dataset 3: BindingDB					
LR	0.887±0.002	0.557±0.015	0.741±0.013	0.896±0.011	0.394
DNN	0.908±0.003	0.613±0.015	0.769±0.028	0.914±0.021	0.371
GNN-CPI	0.900±0.004	0.578±0.015	0.754±0.015	0.903±0.011	0.406
DeepDTI	0.844±0.002	0.429±0.005	0.651±0.024	0.895±0.023	0.060
DeepDTA	0.913±0.003	0.622±0.012	0.780±0.035	0.915±0.016	0.305
DeepConv-DTI	0.908±0.004	0.611±0.015	0.781±0.015	0.905±0.013	0.318
MolTrans	0.914±0.001	0.622±0.007	0.797±0.005	0.896±0.007	0.355

*Note*: MolTrans achieves the best predictive performance across all datasets. The bold value corresponds to the best performance method for each metric.

### 3.4 Q2: MolTrans has competitive performance in unseen drug and target setting

To imitate the unseen drug/target task, we randomly select 20% drug/target proteins and all DTI pairs associated with these drugs and targets as the test set. The results are in Table 3. We observe that KronRLS’s performance vary across settings. This is because KronRLS is a similarity-based method; hence, it is susceptible to the data properties in hand. In the unseen drug setting, we find the one-layer LR is better than multi-layers DNN, and is worse than the SOTA methods with more complicated deep model design. This shows the necessity for carefully designed model architecture. We also see that MolTrans has competitive performance against the SOTA deep learning baselines in both settings.

**Table 3.** MolTrans has competitive result in both unseen drug and protein settings (shown avg. ROC-AUC of five random runs) on BIOSNAP dataset

Settings	DeepDTI	DeepDTA	DeepConv-DTI	MolTrans
Unseen drugs	0.843 ± 0.003	0.849 ± 0.007	0.847 ± 0.009	0.853±0.011
Unseen proteins	0.759 ± 0.029	0.767 ± 0.022	0.766 ± 0.022	0.770±0.029

*Note*: The best performing three baselines are used for comparison.

### 3.5 Q3: MolTrans performs best with scarce data

Although the availability of DTI data is exploding, in some real-world drug discovery pipelines, there are new target proteins or drugs that have only a handful of labels due to budget restriction. Hence, a robust performance under low resource constraint is ideal in DTI setting. We trained each method on 5%, 10%, 20% and 30% of dataset and predict on the rest of them (we use 10% of the test edges as validation set for early stopping). The result is reported in Table 4. We see that MolTrans is the most robust method. In the contrast, SOTA baselines such as DeepDTI and DeepConv-DTI drop as missing fractions increase. One reason why MolTrans is good on scarce setting is that MolTrans leverages on embeddings from sub-structures which are relatively abundant hence transferable compared to other methods which utilize the entire drugs and proteins.

**Table 4.** MolTrans provides best result in high fraction of missing data (shown avg. ROC-AUC of five random runs)

Settings (%)	DeepDTI	DeepDTA	DeepConv-DTI	MolTrans
70	0.853 ± 0.004	0.838 ± 0.004	0.845 ± 0.003	0.853±0.004
80	0.828 ± 0.007	0.821 ± 0.008	0.825 ± 0.003	0.832±0.003
90	0.767 ± 0.010	0.787 ± 0.011	0.792 ± 0.004	0.802±0.004
95	0.659 ± 0.011	0.762 ± 0.004	0.726 ± 0.008	0.768±0.005

*Note*: The best performing three baselines are used for comparison.

### 3.6 Q4: MolTrans is robust in various protein families

**Fig. 2.**
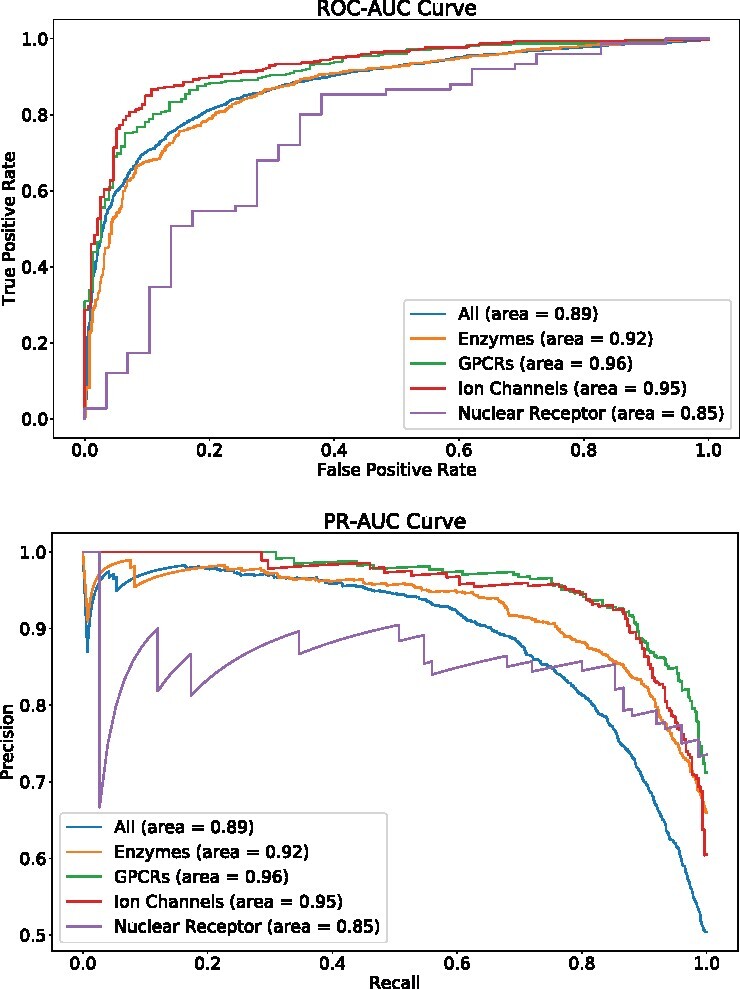
MolTrans is robust in different protein families

Target proteins come from different proteins families. It is important that the prediction algorithm is not biased toward one particular protein family. In this experiment, we test on the predictive performance on four of the largest druggable targets: enzymes, ion channels, G-protein-coupled receptors (GPCRs) and nuclear receptors. We retrieve one test set of BIOSNAP and map the target proteins to the four protein families using GtoPdb database (https://www.guidetopharmacology.org/targets.jsp). We find 1908 enzymes interactions, 533 GPCRs interactions, 496 ion channels interactions and 104 nuclear receptors interactions. We find MolTrans is robust in all of the above individual protein family (Fig. 2). Particularly, enzymes, GPCRs and ion channels have higher performance than the overall protein classes.

### 3.7 Q5: MolTrans allows model understanding

To answer Q5, we show through examples how the interaction map **I** can provide hints on which sub-structure leads to the interaction. A high value cell in the interaction map stands for a potentially activated interaction between drug and target sub-structure that is important to the final interaction outcome. Thus, to visualize, we generate a heat map for **I** to see which cells have high values. We then select a threshold to mask out the majority of cells that have low values. We then examine literature to see if the remaining cells contain clues to the interaction outcome.

**Fig. 3.**
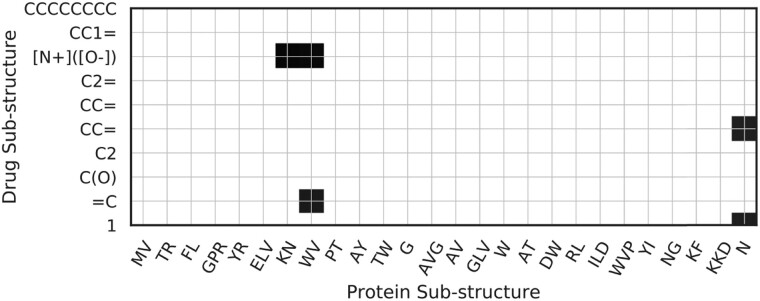
The interaction map on the contributions of sub-structures in DTI, shown as drug 2-nonyl n-oxide interacts with protein cytochrome b-c1 complex unit 10

We first feed drug 2-nonyl n-oxide, and the protein cytochrome b-c1 complex unit 1 into MolTrans, and we visualize the interaction map by filtering scalars that are larger than a threshold in Figure 3. We saw the nitrogen oxide group [N+]([O−]) and KNWV has the highest interaction coefficient, matching with the previous study (Lightbown and Jackson, 1956) who showed that nitrogen oxide group is essential for cytochrome inhibition activity. This example supports that MolTrans is capable of providing reasonable cues for understanding the model prediction and possibly shed light on the inner workings of DTI. To add more credibility, we feed Ephrin type-A receptor 4 (Epha4) target and Dasatinib drug into MolTrans, the map shows amino-thiazole group [S(=O)(=O) and N sub-structures] is highlighted with protein motif KF and DVG, which has an overlap with the Epha4–Dasatinib complex described in previous study (Farenc *et al.*, 2011). We also feed the input target protein HDAC2 and the input drug hydroxamic acid. The interaction map assigns the NC(=O) group and the carbon chain with protein sub-structure KK, YG, DIG, DD with high intensity. The suggested ligand sub-structure matches with the observed interaction in HDAC2-SAHA co-complex (Lauffer *et al.*, 2013). The interaction maps for the additional examples are provided in Supplementary Material S3.

### 3.8 Q6: Ablation study

We conduct an ablation study on the full data setting with the following setup:

-CNN: we remove the CNN from interaction module, and flatten the interaction map **I** output and feed into the decoder.
-AugEmbed: we remove the transformer in the augmented embedding module and feed the interaction module with the positional and content embedding.
-Interaction: we further remove the interaction module from -AugEmbed. It degenerates to a decoder on top of the FCS fingerprint. Note that removing the interaction module alone is not a valid model design.
4. Small: we use smaller dataset to train FCS: DrugBank for drug and BindingDB for protein. We adjust the minimal frequency to output a similar number of sub-structured as FCS-large.
-FCS: we replace FCS embedding with ECFP4 fingerprint for drug and PSC descriptor for protein. The rest of the models remains the same, i.e. they are then fed into transformers, interaction module and decoder.

From Table 5, we see CNN, transformers and interaction module contribute to the model final performance. The FCS fingerprint alone has strong predictive performance from -Interaction. In addition, from Small, we see the massive unlabeled data are useful as it enriches the input and boosts the performance. From -FCS, we see our model is adaptable to other popular fingerprints with similar strong performance.

**Table 5.** Ablation study (five random runs)

Setup	ROC-AUC	PR-AUC
MolTrans	0.895±0.002	0.901±0.004
−CNN	0.876 ± 0.003	0.883 ± 0.006
−AugEmbed	0.876 ± 0.004	0.870 ± 0.004
−Interaction	0.847 ± 0.003	0.859±0.005
Small	0.888 ± 0.001	0.888 ± 0.007
−FCS	0.887±0.004	0.887 ± 0.004

## 4 Conclusion

In this work, we introduce MolTrans, an end-to-end biological inspired deep learning-based framework that models DTI process. We test under realistic drug discovery setting and evaluate with state-of-the-art baselines. We demonstrate empirically that MolTrans has competitive performance in accurately predicting DTI under all settings with an improved explainability.

*Financial Support*: This work was in part supported by the National Science Foundation award SCH SCH-2014438, IIS-1418511, CCF-1533768, IIS-1838042, the National Institute of Health award NIH R01 1R01NS107291-01 and R56HL138415, and IQVIA.

*Conflict of Interest*: none declared.

## Data availability

All data used in the study are from public resources. BIOSNAP is available at http://snap.stanford.edu/biodata/datasets/10002/10002-ChG-Miner.html; DAVIS is available at http://staff.cs.utu.fi/~aatapa/data/DrugTarget/; BindingDB is available at https://www.bindingdb.org/bind/index.jsp. Data processing scripts are also provided in MolTrans GitHub code repository.

## Supplementary Material

btaa880_Supplementary_Data
