# Supplementary material for: MolTrans: Molecular Interaction Transformer for drug–target interaction prediction
Source: Bioinformatics. 2020 Oct 18;37(6):830–6. doi: 10.1093/bioinformatics/btaa880 (PMC8098026; doi:10.1093/bioinformatics/btaa880)
Supplement: btaa880_Supplementary_Data [file btaa880_supplementary_data.pdf]

Supplementary Materials to MolTrans: Molecular Interaction Transformer for Drug Target Interaction Prediction

Kexin Huang[1], Cao Xiao[2], Lucas M. Glass[2], and Jimeng Sun[3]

[1]Health Data Science, Harvard T.H. Chan School of Public Health, Boston, MA
[2]Analytic Center of Excellence, IQVIA, Cambridge, MA
[3]Department of Computer Science, University of Illinois at Urbana-Champaign, Urbana, IL

August 24, 2020

1 Notation Table

Table 1: Main notations used in MolTrans.

Notations	Description
$\mathbf{S}, \mathbf{A}$	drug SMILES, protein amino acids
$\mathcal{I}, \mathcal{U}$	the set of interacting/non-interacting DTI pairs
$\mathscr{C}_p, \mathscr{C}_d$	entire sub-structures set for protein and drug
$\mathbf{C}_\mathrm{p}, \mathbf{C}_\mathrm{d}$	sub-structures set in one pair of drug-target
$\mathbf{M}^\mathrm{p} \in \{0,1\}^{k \times \Theta_p}$	one-hot input representation for protein
$\mathbf{M}^\mathrm{d} \in \{0,1\}^{j \times \Theta_d}$	one-hot input representation for drug
$\mathbf{E}^\mathrm{p} \in \mathbb{R}^{\vartheta \times \Theta_p}$	latent representation for protein
$\mathbf{E}^\mathrm{d} \in \mathbb{R}^{\vartheta \times \Theta_d}$	latent representation for drug
$\mathbf{I} \in \mathbb{R}^{k \times l \times \Psi}$	the interaction tensor
$\mathrm{F}; \mathbf{P} \in [0,1]$	the interaction function; interaction probability
$\mathbf{O} \in \mathbb{R}^{\varphi}$	the output of interaction module
$\mathbf{W}^\mathrm{p}_\mathrm{cont} \in \mathbb{R}^{\vartheta \times k}$	the weight for content embedding protein
$\mathbf{W}^\mathrm{d}_\mathrm{cont} \in \mathbb{R}^{\vartheta \times l}$	the weight for content embedding drug
$\mathbf{W}^\mathrm{p}_\mathrm{pos} \in \mathbb{R}^{\vartheta \times \Theta_p}$	the weight for position embedding protein
$\mathbf{W}^\mathrm{d}_\mathrm{pos} \in \mathbb{R}^{\vartheta \times \Theta_d}$	the weight for position embedding drug
$\mathbf{W}_\mathrm{o}, \mathbf{b}_\mathrm{o} \in \mathbb{R}^{\varphi \times 1}$	the weight, bias for decoder

2 FCS is robust against dataset characteristics

We want to show FCS generate similar set of sub-structures independent of the type of dataset. In this experiment, we generate two set of sub-structures for protein and drug respectively. For protein, one set uses proteins associated with homo sapiens and another uses all proteins in the Uniprot dataset. For drug, one set

uses all drugs in the DrugBank database whereas the other set uses all chemicals in the ChEMBL database. We then compare sets and calculate how many sub-structures are shared. For proteins, we find 32.1% of sub-structures from the homo sapien set is in the Uniprot set. For drugs, we find 38.4% of sub-structures from the Drug Bank set is in the ChEMBL set. Note that since homo sapiens and DrugBank are small datasets, there are noise for FCS to identify the most frequent patterns. Thus, we also calculate the number of sub-structures in the human/DrugBank dataset that have only one character difference in the start/end of sub-structures in the Uniprot/ChEMBL dataset. For proteins, we find additional 54% of these sub-structures (in total, 84.1% similar sub-structures set). For drugs, we find additional 20.2% of them (in total, 58.6% similar sub-structures set). This result suggests that a large portion of FCS generated sub-structures are shared given different dataset characteristics and FCS is not biased against different types of organisms/non-druggable chemicals in the large dataset.

3 FCS's Relation with PrefixSpan

PrefixSpan is for sequential pattern mining, whose sole aim is to find all of the frequent sub-sequences given a set of sequences. In our case, in addition to find these frequent patterns, we also want each sequence to be decomposed into an ordered sequence of discovered frequent subsequences. These subsequences are partitions of the original sequence, which means 1. the union of these subsequences would recover every element in the original sequence; 2. each subsequence is exclusive to each other and should not be overlapped. The decomposition step is not discussed in existing algorithms such as PrefixSpan but is solved by Byte Pair Encoding (BPE) algorithm, which is what FCS is based on. We believe with additional engineering, PrefixSpan can also be extended to the task at hand, but since BPE is already very efficient (took less than 10 minutes for 1.87 millions drugs in ChEMBL), we directly apply BPE here. Note that this partition decomposition step is essential for explainability since an overlapped subsequence fingerprint gives multiple scores for the same subsequence, which leads to ambiguity. For example, suppose a fake sequence CCC1NHCCSCC and C, NH, CCC1, CCC1NH and NHCCS are all frequent patterns from a drug dataset. FCS would decompose it into CCC1 and NHCCS and each of these are given one score, thus a tractable path to see if this substructure is important or not. On the contrary, without the partition requirement, we can represent C, NH, CCC1, CCC1NH and NHCCS for this sequence since they are all frequent. However, if the explanability module scores 0.2 for C, 0.3 for NH, 0.8 for CCC1NH, 0.9 for CCC1, 0.2 for CCC1NH, then, it is hard to know which sub-structure is important. This is also the issue with the popular PubChem fingerprint, which maps each drug into a set of frequent but heavily overlapped substructures.

4 MolTrans is not biased against sequence length

We put the drugs and proteins in the BIOSNAP test set into five bins depending on their sequence length respectively. Then, we calculate the ROC-AUC within each bin. The standard deviation of ROC-AUC is 0.033 for target and 0.038 for drugs, showing that MolTrans is stable in different sequence length.

5 Additional Interaction Feature Maps

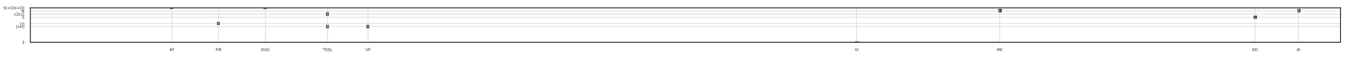

Figure 1: Interaction map of Epha4.

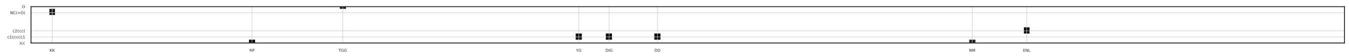

Figure 2: Interaction map of HDAC2.
